# Antioxidant Defense and Pseudoexfoliation Syndrome: An Updated Review

**DOI:** 10.3390/medsci10040068

**Published:** 2022-12-14

**Authors:** Stylianos Mastronikolis, Konstantinos Kagkelaris, Marina Pagkalou, Evangelos Tsiambas, Panagiotis Plotas, Constantinos D. Georgakopoulos

**Affiliations:** 1 Department of Ophthalmology, Medical School, University of Patras, 26504 Patras, Greece; 2 Department of Neurosurgery, James Cook University Hospital, Middlesbrough TS4 3BW, UK; 3 Department of Chemistry, University of Crete, 71500 Heraklion, Greece; 4 Department of Cytology, 417 VA Hospital (NIMTS), 11521 Athens, Greece; 5 Laboratory of Primary Health Care, School of Health Rehabilitation Sciences, University of Patras, 26504 Patras, Greece

**Keywords:** antioxidants, oxidative stress, eye diseases, pseudoexfoliation syndrome

## Abstract

Oxidative stress (OS) affects the anterior ocular tissues, rendering them susceptible to several eye diseases. On the other hand, protection of the eye from harmful factors is achieved by unique defense mechanisms, including enzymatic and non-enzymatic antioxidants. The imbalance between oxidants and antioxidants could be the cause of pseudoexfoliation syndrome (PEXS), a condition of defective extracellular matrix (ECM) remodeling. A systematic English-language literature review was conducted from May 2022 to June 2022. The main antioxidant enzymes protecting the eye from reactive oxygen species (ROS) are superoxide dismutase (SOD), catalase (CAT) and glutathione peroxidase (GPx), which catalyze the reduction of specific types of ROS. Similarly, non-enzymatic antioxidants such as vitamins A, E and C, carotenoids and glutathione (GSH) are involved in removing ROS from the cells. PEXS is a genetic disease, however, environmental and dietary factors also influence its development. Additionally, many OS products disrupting the ECM remodeling process and modifying the antioxidative defense status could lead to PEXS. This review discusses the antioxidative defense of the eye in association with PEXS, and the intricate link between OS and PEXS. Understanding the pathways of PEXS evolution, and developing new methods to reduce OS, are crucial to control and treat this disease. However, further studies are required to elucidate the molecular pathogenesis of PEXS.

## 1. Introduction

**Figure 1:**
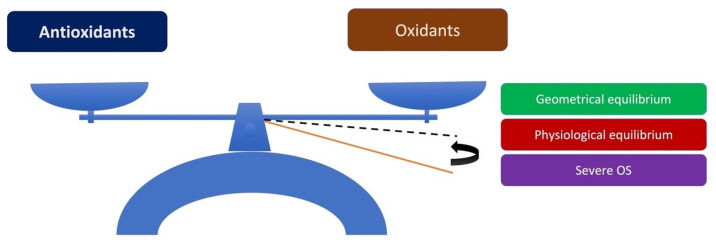
Model of normal and disturbed antioxidant/oxidant equilibrium. The physiological equilibrium is represented by a black dashed line, while the severe OS is represented by a solid orange line.

Oxidative stress (OS) is defined as excess reactive oxygen species (ROS) production, mainly because of the imbalance between the generation and clearance of free radicals and reactive metabolites. Generally, OS results from ROS overproduction or insufficient antioxidant defense. The average balance between ROS production and antioxidant protection is slightly towards the oxidation products side, thus favoring a mild OS. Such a balance prevents the accumulation of damage but also allows the existence of a sufficient number of ROS to perform cellular signaling functions. Severe equilibrium disruption towards the produced ROS’s side can lead to intense OS (Figure 1). The delicate balance between ROS’s beneficial and harmful effects is critical to living organisms and is maintained by “redox regulation”. The redox regulation maintains homeostasis and protects living organisms from OS. Importantly, OS is essentially a disorder in the redox regulation [1]. Excessive ROS production causes OS in the cells, resulting in cell damage. Thus, cells contain antioxidant defense mechanisms to neutralize the excess ROS and maintain the redox balance, which is vital for cell survival [2]. Studies on OS in the post-excimer cornea laser found that re-epithelialization had considerably accelerated when free radicals were reduced [3,4,5,6]. These studies have implemented: (i) oral cysteine supplementation in a daily dose of 200 mg [3], (ii) cysteine 5 mg 5 microg/10 microl phosphate-buffered saline 3 times a day for 1 week [6], (iii) topical basic fibroblast growth factor 10 μg per 10 μL four times a day for 7 days plus oral L-cysteine supplements 500 mg once a day for 15 days [4], and (iv) basic fibroblast growth factor eyedrops 5 microg/10 microl phosphate-buffered saline 3 times a day for 1 week after excimer photoablation [5]. Overall, when there is a disturbance of antioxidants/oxidants equilibrium, the resulting OS can induce many pathologies in almost every organ in the human [7]. This review discusses the antioxidative defense of the eye in association with pseudoexfoliation syndrome (PEXS).

Cumulative data support the assumption that chronic stress conditions, especially OS, contribute significantly to pathogenesis and abnormal matrix accumulation in the context of PEX syndrome/glaucoma [8]. OS results from an imbalance between damaging oxidants, such as ROS or nitrogen molecules, and protective antioxidants, such as enzymes (e.g., superoxide dismutase (SOD), glutathione peroxidase (GPx), catalase (CAT)), vitamins, glutathione (GSH), uric acid, etc., as well as impaired repair mechanisms for oxidative damage to proteins, lipids and nucleic acids. Decreased antioxidant levels and increased concentrations of degradation products of oxidatively damaged biomolecules (e.g., malondialdehyde (MDA)) are, therefore, considered reliable markers of OS [9]. Antioxidant protection enzymes are downregulated at the mRNA level in tissues of the anterior segment of the eye in individuals with PEX, as has been demonstrated primarily for several isoforms of glutathione S-transferase (e.g., GST-M1, GST-T1) [10,11]. Compared to age-appropriate controls, the oxidant–antioxidant balance in both aqueous humor (AH) and serum of PEX patients was shifted in favor of oxidants. OS is prominently involved in the progression of the syndrome as it promotes fibrogenesis via the induction of transforming growth factor β (TGF-β) and numerous bimolecular effectors involved in the dysregulation of extracellular matrix (ECM) [12]. Increased concentrations of fibrogenic growth factors (e.g., TGF-β1), decreased activities of proteolytic enzymes ((e.g., matrix metalloproteinase 2 (MMP-2)), subclinical inflammatory processes and increased cellular stress are causally involved in this abnormal matrix process [8]. Etiologically, this fibrotic process seems to result from an interplay of genetic and environmental factors in the sense of a complex disease [9,13]. Recent genetic studies have shown significant associations of PEX patients with polymorphisms in OS-related genes such as lysyl oxidase-like 1 (LOXL1) and clusterin (CLU) [14,15,16,17]. While short-term stress exposure evokes an adaptive cellular stress response, the aim of which is to restore homeostasis, in the case of prolonged chronic stress conditions, the repair mechanisms of the cells are overwhelmed, which sooner or later results in irreparable pathological tissue changes [18]. Chronic stress conditions, accompanied by successively overtaxed cellular repair mechanisms, lead to the chronic activation of molecular inflammatory agents, an induction of the pro-fibrotic growth factor TGF-β1, stimulation of the extracellular matrix synthesis as well as accelerated cell aging and cell loss, which are centrally involved in the pathophysiology of the PEX-specific matrix process in meditating the degenerating fibrotic process. In fact, recent findings have shown that early PEX has significantly increased concentrations of the pro-inflammatory cytokines interleukin (IL)-6 and IL-8 in AH and increased mRNA expression rates in tissues of the anterior segment of the eye [9,19,20]. In vitro models have confirmed that IL-6 and IL-8 are affected by OS and hypoxia. The pro-inflammatory mediators themselves can induce numerous disease-relevant key molecules such as TGF-β1, LOXL1, tissue inhibitors of metalloproteinases (TIMPs) and elastic fibrous proteins. TGF-β1, a potent profibrotic growth factor, then causes a whole series of molecular processes that eventually lead to abnormal matrix accumulation and tissue fibrosis, i.e., the accumulation of PEX material. TGF-β-induced matrix enrichment in the trabecular meshwork is ultimately responsible for the progressive increase in outflow resistance and is directly related to intraocular pressure levels and glaucomatous optic nerve damage in pseudoexfoliation glaucoma (PEXG) [12,15,18,21,22,23,24,25]. Moreover, disorders in the oxidative balance, including increased levels of selected oxidants, occur in PEXS and, consequently, PEXG. Under these conditions, increased levels of MDA, which is involved in lipid oxidation, were observed. Simultaneously, the levels of antioxidant enzymes decrease [12,25,26,27,28]. Numerous studies have documented an altered oxidant–antioxidant balance in AH, serum and ocular tissues of PEX patients [8,9,11,16,20,21,24,29,30,31,32,33,34,35,36,37,38,39,40,41,42,43,44,45,46,47,48,49,50,51,52]. The Reykjavik Eye Study, a large population-based epidemiological study, showed that, in addition to age and gender, individual antioxidant status is a significant risk factor for PEX development [53]. Regarding PEXG, a 6-month open-label randomized trial showed that oral docosahexaenoic acid (DHA) supplementation ameliorated intraocular pressure (IOP) in patients in the experimental group [54]. Total antioxidant capacity (TAC), paraoxonase (PON) and arylesterase (ARE) levels in AH and serum of the PEXS and PEXG patients were significantly decreased compared with the control group (*p* < 0.05). Toluenesulfonyl group (TOS) values were higher in patients with PEXS and PEXG than in controls (*p* < 0.05) [55]. The consumption of dietary products with high folate content lower homocysteine (Hcy) levels, which reduces the risk of PEXG [56]. Serum samples were examined using spectrophotometric and enzymatic methods, and the TAC was assessed in individuals with PEXG. Reduced levels of antioxidants were observed in the serum samples of patients compared to those of controls, which may indicate the involvement of OS and the significance of antioxidants in the pathogenesis of PEXG [40]. Disturbances in the oxidative balance have been linked to the occurrence of PEXG in the course of PEXS [16].

PEXG may manifest unilaterally or bilaterally, similarly to other secondary glaucomas (e.g., pigment dispersion, neovascular and inflammatory glaucomas). Unilateral cases may become bilateral over time (progression to bilaterality in up to 50% of patients within 5 to 10 years after diagnosis), as this is a systemic disease that increases in severity with age [57,58]. Recent studies have demonstrated that PEXS is a systemic process with a wide distribution of PEX material deposits in the body, including the skin, meninges, lungs, heart, and other visceral organs. In accordance, typical PEX accumulations have been detected by electron microscopy in the conjunctiva and in other peribulbar tissues of clinically involved and virtually all uninvolved fellow eyes. Another light microscopic immunohistochemical study demonstrated abnormal deposits similar to those of classic PEXG in the periphery of iris vessels of clinically unaffected eyes. Together, these findings indicate that so-called unilateral PEXG is clinically asymmetric rather than truly unilateral. Since both eyes are obviously affected by the PEX process, the term unilateral PEXG, which is most common in clinical practice, is actually misleading, and the PEXS is probably never truly unilateral. The reasons for this marked asymmetry remain largely unknown, but it may be influenced by local modulating factors, such as imbalances of growth factors and oxidative stress, or by subtle differences in hemodynamics or aqueous humor dynamics between both eyes. Genetic factors are now considered as predisposing factors for PEXG [59,60].

## 2. Materials and Methods

A systematic review of the literature published in English was performed from May 2022 to June 2022 to identify all published reports on OS and antioxidant defense in pseudoexfoliation syndrome in the eye. Studies were identified by combination research of Cochrane, Scopus and PubMed (National Library of Medicine, Bethesda, MD, USA) databases between January 1952 and May 2022. The following keywords and MeSH terms were used “antioxidants”, “pseudoexfoliation”, “pseudoexfoliation syndrome”, “pseudoexfoliation material”, “oxidative stress”, and “eye”.

## 3. Results

### 3.1. Disturbance of Antioxidant/Oxidant Equilibrium in the Pathogenesis of PEXS

**Figure 2:**
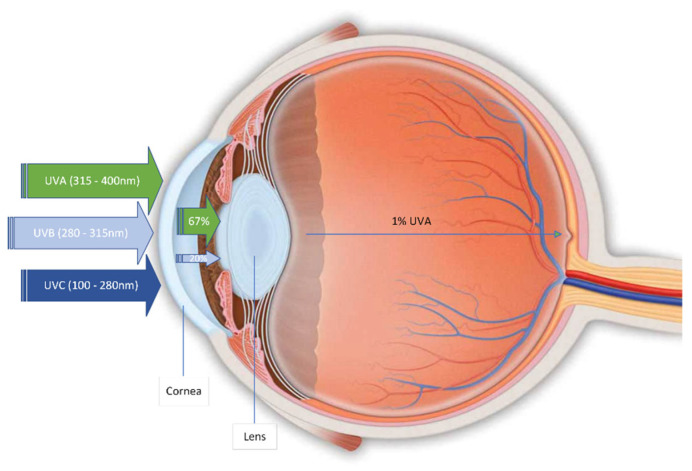
Solar UVR absorption by the eye elements.

OS affects the structural characteristics of anterior eye tissues, rendering them susceptible to several risk factors, including environmental factors, such as the sun (Figure 2).

**Figure 3:**
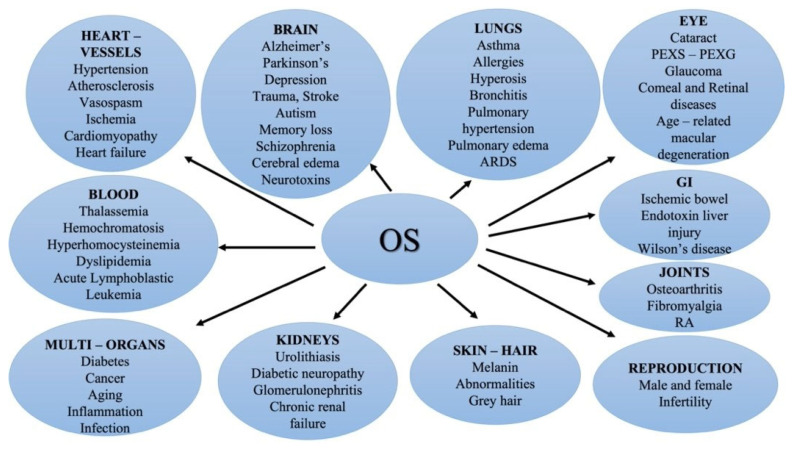
Organic systems and human diseases associated with OS.

Studies indicate that almost every component of the eye is susceptible to OS. As shown in Figure 3, OS is mainly involved in the etiology of several ophthalmic diseases, such as cataracts, PEXS/PEXG, glaucoma, age-related macular degeneration (AMD), diabetic and light-induced retinopathy, retinal degeneration and corneal diseases, and systemic disorders [61,62]. The PEXS primary impact locus has been identified as LOXL1 (lysyloxidase-like 1). However, every known common variation related to PEX exhibits an allele effect reversal among populations with various ancestries, raising questions about their biological importance. In nine different ethnic ethnicities, rs7173049A>G, a frequent non-coding sequence variation downstream of LOXL1, was consistently linked to a lower incidence of PEX (odds ratio, OR = 0.63; *p* = 6.33 10–31). The protective rs7173049-G allele is associated with higher tissue levels of the immunoglobulin superfamily containing leucine-rich repeat 2 (ISLR2) and signaling receptor and transporter of retinol (STRA6), and these genes, as well as other essential elements of the STRA6 receptor-driven retinoic acid (RA) signaling cascade, are markedly downregulated in tissues of PEX patients. In PEX-relevant cell types, the siRNA-mediated downregulation of RA signaling results in the overexpression of LOXL1 and PEX-associated matrix genes. These findings show that the pathophysiology of PEXS is influenced by the dysregulation of STRA6 and impaired retinoid metabolism, and that the variant rs7173049-G, the first common variant at the broad LOXL1 locus without allele effect reversal, mediates a protective effect by upregulating STRA6 in ocular tissues [63]. Additionally, the rs11638944:C>G transversion has a cis-acting effect on LOXL1 expression levels through modulating alternative splicing of LOXL1 and differential binding of the transcription factor RXR (retinoid X receptor alpha), which ultimately results in lower levels of LOXL1 mRNA in the cells and tissues of risk allele carriers. These results reveal a functional mechanism by which frequent non-coding mutations affect the expression of LOXL1 [23].

Supportive evidence points out that malondialdehyde (MDA), a marker of free-radical-mediated lipid peroxidation, is high in the patients of PEXS [64]. Similarly, thiobarbituric acid reactive substances (TBARS), another oxidative marker, were also significantly higher in the aqueous humor (AH) samples collected from primary open-angle glaucoma patients [65]. Besides, the levels of advanced glycation end products (AGEs) are also very high in the AH of PEXS patients [9,25]. Emerging evidence indicates that these specific oxidation and glycation products could trigger the glaucoma formation associated with PEXS [66]. Specifically, these end products might induce ROS generation, thus causing damage to trabecular meshwork cells [67,68,69]. These findings indicate that OS might play a crucial role in the pathogenesis of PEXS.

Another possible way the OS could be involved in the pathogenesis of PEXS is via elevating the levels of free radicals and transforming growth factor β (TGF-β) in the eye, which is vital for developing fibrosis in the PEXS-affected eyes [37]. Additionally, OS disrupts the homeostatic balance between matrix metalloproteinases (MMPs) and their tissue inhibitors (TIMPs), causing dysregulated ECM in PEXS patients [22]. Further evidence indicates the synergy between TGF-β1 and OS in activating LOXL1, suggesting OS might regulate the ECM homeostasis via both TGF-β1 and LOXL1 [70].

Further, OS is also known to influence the activity of the enzyme glutamine synthase and modulate glutamate/glutamine metabolism, leading to increased accumulation of glutamate [71]. Besides, OS also damages the mitochondria of optic nerve cells, leading to reduced energy production in these cells [72]. In addition, OS can impair vascular functions, resulting in reduced blood flow to the optic nerve, injury to the trabecular meshwork, elevated IOP and glial cell dysfunction [28,73]. Overall, these findings unambiguously pinpoint OS as one of the master regulators of PEXS pathogenesis.

Measurements from the serum, AH and lens tissues of PEXS/PEXG patients demonstrate that the PEXS-affected eye is constantly increasing OS with impaired antioxidant protection [41,44]. The levels of oxidizing molecules such as hydrogen peroxide (H_2_O_2_) and nitric oxide (NO), TOS and other OS biomarkers such as lipid peroxidation products, degradation products of methylated and oxidized proteins and protein carbonyl groups, Hcy and AGEs, are significantly increased in eye tissues and serum. On the contrary, the concentrations of essential antioxidants such as ascorbic acid, GSH, trace elements and antioxidant enzymes, and the TAC in samples of PEXS/PEXG patients, are significantly reduced, indicating a defective antioxidant defense system and insufficient cytoprotection against OS. Various studies have reported an essential increase in serum and aqueous TOS and a substantial decrease in serum TAC levels in PEX patients [41]. Further, 8-Iso-PGF_2a_ and NO AH levels are highly increased, and ascorbic acid significantly decreased in PEXS patients [29,35]. Similarly, the proinflammatory cytokine tumor necrosis factor (TNF-α) and NO levels were significantly higher in PEXG patients [74].

On the contrary, serum NO concentration was significantly lower in PEXG compared to PEXS patients. These discrepancies could be due to the changes in NO possibly involved in the evolution from PEXS to PEXG by regulating the vascular flow to the eye [75]. Further studies found significantly higher levels of high-sensitivity C-reactive protein (CRP) and TNF-α in the serum of PEXS patients [76]. Similarly, early PEXS patients exhibited significantly higher interleukin-6 (IL-6) and interleukin-8 (IL-8) levels in the AH, suggesting a pathogenic role of pro-inflammatory cytokines in the early and late stages of PEXS/PEXG patients [20]. Studies indicate asymmetric dimethylarginine (ADMA) and its structural isomer, symmetric dimethylarginine (SDMA), as possible PEXS biomarkers [77]. PEXS was associated with increased ADMA levels of serum and aqueous humor, as well as growth factors, MMPs, TIMPs, and endothelin-1 (ET-1) [36]. Likewise, MMP-2 and -3 and TIMP-1, -2 and -4 and ET-1 were detected at significantly higher concentrations in AH samples from PEXS/PEXG eyes [78,79]. Along these lines, high levels of TGF-β1, IL-8, macrophage inflammatory protein-1α (MIP-1α), fractalkine, and the immune cell growth factor Flt3 ligand were observed in the AH of PEXG patients. The higher levels of cytokines induced by OS might regulate ECM remodeling, IOP elevation, and PEXG field pathogenesis [39].

Several studies have attempted to determine the role of thiol–disulfide in PEXS [80]. The serum thiols mainly consisted of albumin and thiols in other proteins and a small fraction of low-molecular-weight thiols such as GSH, glutathione disulfide (GSSG), cysteine, Hcy, cysteinyl glycine and γ-glutamylcysteine [81]. Because of their reactivity, thiols are considered a key target for oxidation. Thus, cysteine residues can form disulfide bonds between protein and low-molecular-thiol groups under OS conditions, especially with S-glutathionylation [82]. Therefore, thiol–disulfide homeostasis is vital in the antioxidant defense system and controls the molecular responses to oxidants. High concentrations of GSSG and a decreased ratio of GSH/GSSG have been found in the AH of patients with PEXS and cataracts, with increased MDA levels and reduced GSH levels in PEXS lens epithelial cells in comparison with non-PEXS analogous cells [83]. Nevertheless, a significant increase in serum GSH levels in patients with PEXS and PEXG was also noted. However, the mean total serum thiol, native thiol levels, and serum native thiol/disulfide ratio were significantly lower. In contrast, the mean serum disulfide level was considerably higher in PEXS patients than in healthy controls [47]. Recent reports also suggest that total thiol and native thiol levels were higher in the PEXG [48]. Protein carbonyls have also been measured in serum and AH. Their mean concentration was much higher in PEXS patients than that of controls. Measurement of carbonyl groups is considered a good marker of the degree of OS in the proteins [34].

Further, the levels of superoxide dismutase 2 (SOD2), aldehyde dehydrogenase 1 (ALDH1A1), and microsomal glutathione S-transferase 1 (MGST1), which are part of the essential antioxidant defense system, are high in the anterior lens capsule of PEXS patients [84]. In contrast, antioxidant enzymes, SOD and CAT were significantly lower in PEXS patients [25]. Similarly, the levels of GSH decrease in the lens epithelial cells of PXES patients [26,83]. MGST1 could be a protective factor against lipid peroxidation OS. Thus, a significant increase in the mRNA level may play a role in PEXS development [85,86,87]. Besides, low MGST1 and CLU expression levels could be an early indicator of OS-related PEXS [88]. Other studies found reduced serum ascorbic acid concentrations. Compared to controls, the increased MDA levels in PEXS and PEXG patients suggest that lipid peroxidation is an essential factor in their pathogenesis and evolution from PEXS to PEXG [25]. ALDH1A1 is an enzyme that participates in the oxidation of various aldehydes and the metabolism of lipid peroxidation products such as MDA [89], and thus plays a crucial role in preventing ROS formation [90]. It is believed that the antioxidant defense failure could result in inadequate OS response and PEXS development.

A notable decrease in serum SOD activity in PEXS patients compared with controls was also reported, in contrast to significantly higher MDA and carbonyl protein levels [25,34]. While SOD demonstrated increased activity in the lens capsule of PEXS patients with cataracts compared to patients with cataracts alone, it supports the role of OS in the pathogenesis of PEXS and cataracts. Serum CAT also exhibited significantly lower catalytic activity in PEXS patients suggesting a deficiency in the enzymatic antioxidant protection system [91]. A crucial MMP, prolidase, is involved in collagen biosynthesis and matrix remodeling; thus, serum prolidase activity (SPA) could be a candidate for disease pathogenesis. Studies suggest that SPA levels were significantly lower in PEXS and may cause amyloid aggregation in PEXS [27]. Studies also indicate that it reduced PON and ARE activity levels in AH of PEXS/PEXG patients [46]. In contrast, paraoxonase 1 (PON1) activities were significantly higher in PEXS patients [41]. Further studies analyzing any link between three genetic variants of antioxidant enzyme PON1 and PEXS/PEXG suggested a possible role for the PON1 promoter variant in PEXS [92].

In PEXS patients, zinc levels were lower in lens tissues, and selenium levels were lower in serum, AH, and conjunctiva samples [43]. Selenium is an essential structural element of GPx. Along with selenium-dependent enzymes and thioredoxin reductase (TrxR) activity, it is responsible for sustaining the antioxidant capability of vitamin C by catalyzing its regeneration [24]. Several studies investigating the role of selenium in PEXS development exhibited that lower selenium levels in AH, conjunctiva and serum of patients could advocate the concept of an impaired antioxidant defense system [43]. Studies further explored the role of xanthine oxidase (XO), a ROS-producing enzyme in PEXS. Higher aqueous XO levels were found in PEXS patients, suggesting a higher OS in the anterior chamber of PEX patients [11,93].

The protein composition of AH samples in PEXS eyes was recently examined using high-resolution mass spectrometry. Significantly increased levels of complement factor 3 (C3), antithrombin III, kininogen-1 (KNG-1), and vitamin D-binding protein (DBP) and decreased levels of retinol-binding protein 3 (RBP3), glutathione peroxidase 3 (GPX3), carboxypeptidase E (CPE) and calsyntenin-1 (CLSTN1) were observed. These findings suggested that OS and inflammation could contribute to PEXS development [33]. Besides, an increased expression with terminal deoxynucleotidyl transferase-mediated dUTP-biotin nick end labeling (TUNEL) and a decreased expression with proliferating cell nuclear antigen (PCNA) were observed in cataract patients with PEXS, suggesting increased apoptosis and a reduced proliferation of lens epithelial cells [94]. The metabolic profiles of PEXS patients were recently examined. The results suggested significantly lower levels of amino acids and their derivatives, such as arginine and homo-arginine, hydroxy butyryl carnitine and decatrienoylcarnitine in AH samples. Similarly, the antioxidants such as ascorbic acid, hydroxyanthranilic acid and S-adenosylmethionine were also found to be low in AH samples obtained from PEXS patients [95].

### 3.2. OS in the Eye

OS affects the structural characteristics of the anterior eye tissues, rendering them susceptible to several risk factors, including environmental factors that cause OS [8]. The environmental factors harmful to the eye include atmospheric oxygen, solar and cosmic radiation, toxins, air pollution, artificial light, cigarette smoke, toxic gases, drugs and physical injury [96,97]. The radiation from the sun has several components, including ultraviolet radiation (UVR), namely UVA (315–400 nm), UVB (280–315 nm) and UVC (100–280 nm). Studies suggest that UV radiation is the primary source of ROS in the eye [96]. The cornea is exposed directly to UVR and absorbs all of UVC, 80% of UVB and 34% of UVA; the aqueous humor (AH) absorbs some of the UVB, the lens absorbs 66% of UVA and 20% of UVB and the retina absorbs only a minimal percentage of UVA (<1%), but no UVB or UVC (Figure 2). The ocular tissues’ absorption of UVR, especially UVC and UVB, leads to the photochemical production of ROS (e.g., singlet oxygen (^1^O_2_), superoxide (O_2_^•−^), hydroxyl radical (OH^•^), peroxyl radical (ROO^•^)) [97,98]. The result is UVR-induced molecular modifications such as chain-breaking, pyrimidine and thymine dimers and protein crosslinks associated with cataracts, glaucoma and AMD [99,100]. Scandinavians have been found to have a high PEXG prevalence (23%), which has been attributed to genetic susceptibility (LOXL1 gene). Other neighboring nations, also exposed to similar sunshine conditions, do not share the same prevalence rates, for instance, Germans (4.7%) and English (4%). The lowest prevalence of PEXG has been reported in the Greenland population at 0% [12,101,102]. The presence of pseudoexfoliation material has been found to be of multifactorial nature in the basis of ECM dysregulation. In short, old age (rarely occurs below the age of 50), race (Nordic and eastern Mediterranean populations), darker iris pigmentation, environmental factors (e.g., time spent outdoors, altitude) and diet, have been mainly associated with presence of pseudoexfoliation material in the eye [103,104].

In addition, the eye can be affected by OS due to its specific physical and metabolic characteristics. Specifically, the mitochondrial ROS, a byproduct of metabolism, is a significant endogenous source in the eye as it consumes large amounts of O_2_. Further, the transparent features of the eye, specifically the cornea, AH, lens, vitreous and retina, allow for the continuous photochemical production of ROS [61]. Studies indicate that almost every component of the eye is susceptible to OS. As shown in Figure 3, OS is mainly involved in the etiology of several ophthalmic diseases, such as cataracts, PEXS/PEXG (pseudoexfoliation glaucoma), glaucoma, AMD, diabetic and light-induced retinopathy, retinal degeneration and corneal diseases.

#### 3.2.1. OS in the Lens

The lens absorbs over 60% of UVA and 20% of UVB and protects the retina [10]. Continuous exposure to solar UVR and oxidants such as smoke and low levels of antioxidant molecules in the lens predisposes the lens to a higher OS [105]. Further, endogenous lens ROS is also generated by the metabolism. For example, O_2_^•−^ is caused in the electron transport chain by the activity of cytochrome P450. Similarly, the nicotinamide adenine dinucleotide phosphate (NADPH) oxidase complex generates in response to growth stimuli. Further, the intracellular H_2_O_2_ is derived by the action of SOD or from ascorbate and O_2_ in the presence of Fe^3+^. Finally, the reaction of H_2_O_2_ with metal ions can generate O_2_^•−^ or OH^•^ via the Fenton reaction [106]. Under OS conditions, the oxidation of specific amino acids causes cross-linking and aggregation of proteins [107,108]. It also leads to cataract development [109]. To protect itself against OS, the lens contains high concentrations of ascorbic acid and glutathione (GSH) [110]. However, during aging, the GSH levels decline and thus, ascorbic acid is oxidized, causing the accumulation of crystalline-bound advanced glycation end products (AGEs) and cataractogenesis [111]. As a result, the photooxidation of the thiol groups of the lens’ crystallins forms disulfide bridges between the molecules, leading to further protein aggregation and cataract formation [112].

#### 3.2.2. OS in the Lens Epithelium

UVA is the leading cause of ROS generation in the lens epithelial cells [113]. In addition to UV-induced damage, the oxidation of macromolecules of lens epithelial cells is also triggered by excessive cellular oxidants produced by exposure to toxic chemicals [114]. Moreover, lens epithelial cells are also affected by the high levels of H_2_O_2_ produced in the aqueous humor [115]. Studies suggest that the H_2_O_2_ and peroxynitrite (ONOO^−^) are the oxidants of lens epithelial cells [114], and their elevated levels trigger oxidation-dependent inactivation CAT, proteasome, and arylamine N-acetyltransferases (NATs) [114,116]. Oxidative damage to lens epithelial cells causes the lens’s osmotic swelling and results in its transparency loss [117]. Further, exposure of lens epithelial cells to UVA results in lipid peroxidation, decreased antioxidant defense by enzymes and cell death [118]. Similarly, UVB triggers lens epithelial cell apoptosis via the inactivation of antioxidant defense enzymes [114,119].

#### 3.2.3. OS in the AH

UVR and inflammatory processes in adjacent structures are the principal reasons for the OS in the AH [120,121]. It is worth mentioning that the AH contains ascorbic acid, uric acid and amino acids (tyrosine, phenylalanine, cysteine, tryptophan) as antioxidants. These antioxidants absorb most UVR and thus protect the eye from damage [122]. Even with these protective features, photooxidation generates potent oxidizing molecules, such as ^1^O_2_ and H_2_O_2_ [123]. This process reduces GSH metabolism [124], leading to damage to the eye’s structures, including corneal endothelium, the lens and the trabecular meshwork.

### 3.3. Antioxidant Defenses in the Eye

Protection of the eye from harmful external factors is achieved by non-specific defense mechanisms such as eyelids, tear film, cornea, and lens. When these external factors overcome the above barriers, they encounter unique defense mechanisms, including enzymatic and non-enzymatic antioxidants. Antioxidants are endogenous or exogenous substances that work in low concentrations and significantly prevent or delay substrate oxidation in an enzymatic or non-enzymatic reaction. Almost every organism has natural antioxidant defense mechanisms to deal with the harmful effects of free radicals generated in the cells [61]. SOD, CAT, GPx, thioredoxin (Trx), peroxiredoxin (Prx), glutathione S transferase (GST), arylesterase (ARE) and PON are some of the most common enzymes that function as antioxidants [125]. Non-enzymatic antioxidants include vitamins A, C, E, GSH, uric acid, phenolic compounds, melatonin and serum proteins such as ceruloplasmin, albumin and transferrin [61,126]. Depending on their source, antioxidants are divided into endogenous, initially synthesized in the body (e.g., GSH, SOD, CAT), and exogenous, which can be obtained through diet and supplements (e.g., GSH, vitamins C and E, carotenoids, flavonoids and trace elements). Three distinguished levels characterize the antioxidant defense system. The first level prevents the formation of free radicals and consists of enzymes such as SOD, CAT and GPx. The second level suppresses chain initiation and breaks the chain propagation reactions, implicating low-molecular-weight antioxidants, for example, vitamins and enzymes such as ALDH1A1. The third level comprises repair enzymes such as GPx and MGST1 [127].

- Antioxidants in the crystalline lens: Environmental radiation usually does not harm the human lens due to its protective antioxidant system and chromophore. The lens has various protective and repair systems to deal with OS, mainly ascorbic acid and high levels of reduced GS. Lens also contains antioxidant enzymes, such as SOD, CAT, GPx, other antioxidants like the carotenoids named lutein and zeaxanthin, tocopherols, retinoids, and taurine [128]. In addition, α-crystalline, accounting for over 50% of the total protein mass of the mammalian lens, acts as a molecular chaperone that prevents heat-induced aggregation of many proteins and is also required for the renaturation of chemically denatured proteins. α-crystalline has been demonstrated to inhibit protein aggregation in vitro caused by UVR and OS. Thus, it is believed to protect in vivo lens proteins from photooxidation alterations. Finally, a transsulfuration pathway in the lens has been demonstrated and adjusted under OS conditions, offering different redox potential in cells. This pathway can be characterized as a new defense system against OS [128,129].
- Antioxidants in the crystalline lens epithelium: The lens epithelium has a wide range of antioxidant defense mechanisms, such as the antioxidant enzymes SOD, CAT and the GSH and Trx systems. GSH is also found at high levels in the lens and even higher in the lens epithelium. The total glutathione in a healthy lens epithelium is almost entirely in reduced form (GSH). Still, minimal, virtually undetectable, oxidized levels (GSSG) coexisted. In addition, the lens epithelium contains an active glutathione redox cycle, through which GR, NADPH and the hexose monophosphate shunt (HMPS) pathway efficiently reduce GSSG to GSH. The GSH system includes GPx, NADPH, GR, glutaredoxin (Grx) and GSH levels. The target molecules of the lens epithelial cells protected by the glutathione system are specific cytoskeletal proteins and proteins that maintain average membrane permeability and proteins containing critical sulfhydryl groups necessary for normal epithelial functions (e.g., Na⁺/K⁺-ATPase) [130]. The Trx system has a variety of biochemical processes, such as the detoxification of H_2_O_2_, regulation of cell death and activation of transcription factors that regulate cell growth and production of deoxyribonucleotides for DNA synthesis. Both systems can reduce protein disulfide bonds; Trx operates at the micromolar levels and GSH at the millimolar levels. Both systems act selectively on target proteins and metabolic pathways, but the Trx system regulates more proteins and pathways than GSH [131].
- Antioxidants in the AH: The human AH includes non-enzymatic antioxidants such as ascorbic acid (530 μM), L-tyrosine (78 μM), uric acid (43 μM), L-cysteine (14.3 μM) and GSH (5.5 μM). The AH in nocturnal species has a different composition, i.e., the nocturnal rat contains glutathione (125 μM) and L-cysteine (63 μM). The higher concentration of ascorbic acid in the AH of diurnal species compared to that of nocturnal species is a strong indication of the critical protective role of ascorbic acid against UVR. This protection is attributed to direct UVR absorption, fluorescence quenching of biomolecules and control of the fluorescence-mediated biotransformation [132]. L-tyrosine is the second most abundant water-soluble antioxidant in the human AH and is an OH^•^ purifier, ^1^O_2_ quencher and weak photosensitiser. Uric acid is a purine derivative found in the lacrimal layer, AH and other extracellular fluids [133]. This water-soluble molecule has high activity against ^1^O_2_ and OH^•^, serving as a possible purifier. It has also been proposed to regulate the glutathione–ascorbic system’s redox status. L-cysteine replenishes its reservoir in the AH and acts as an antioxidant directly through the thiol group. Unlike other ocular tissues, AH contains minimal amounts of protein and antioxidant enzymes. The activity of SOD in human AH is minimal and probably does not contribute significantly to its overall antioxidant defense [132]. Thus, the defense of the AH is mainly based on the extremely high levels of low-molecular-weight antioxidants such as ascorbic acid. Specifically, the concentration of ascorbic acid in the AH is higher than that in the blood plasma [134].

#### 3.3.1. Enzymatic Eye Antioxidants

The main antioxidant enzymes protecting the eye from ROS are SOD, CAT and GPx, which catalyze the reduction of specific types of ROS [61] (Table 1).

##### SOD

**Figure 4:**
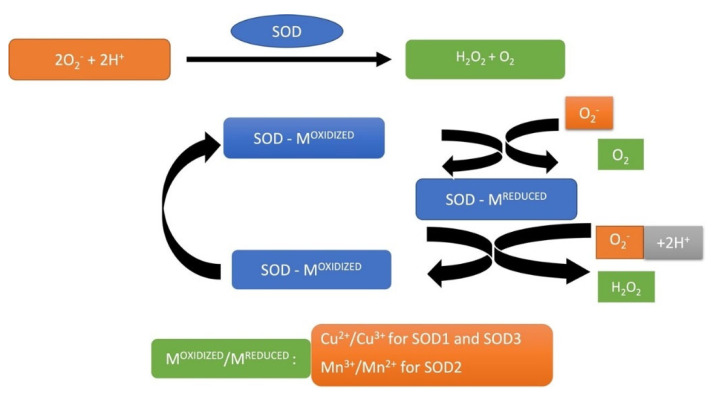
O_2_^•−^ scavenging by SOD with consecutive reduction and re-oxidation of the catalytic metal (Cu or Mn) in the active region of the enzyme.

SOD is a metalloprotein and a critical antioxidant enzyme in ROS metabolism that catalyzes the auto-redox reaction of O_2_^•−^ to H_2_O_2_ and O_2_ (Figure 4). At normal pH, the rate of spontaneous dismutation of O_2_^•−^ is considerable but increases significantly with the SOD. SOD can be detected in three isoforms: (1) the cytoplasmic isoform of copper/zinc (CuZnSOD, SOD1) [135,136]; (2) mitochondrial isoform of manganese (MnSOD, SOD2); (3) extracellular isoform (ECSOD, SOD3) [137,138]. In conjunction with CAT and GPx, SOD plays a protective role in preventing H2O2 from accumulating and converting into highly reactive OH^•^. SOD isoforms have been identified in the lacrimal layer, cornea, AH, iris, radial body, lens and lens epithelium, vitreous, sclera and the retina [45].

##### CAT

Catalases or hydro peroxidases catalyze the conversion of H_2_O_2_ to H_2_O and O_2_. More than 300 isoforms have been found. The majority are the classical heme iron enzymes or monofunctional catalases, catalase-peroxidases or bifunctional catalases and a small group of enzymes containing manganese [139].

CAT has two enzymatic activities depending on the concentration of H_2_O_2_:

At high H_2_O_2_ concentrations, CAT acts as a catalyst removing H_2_O_2_ and forming H_2_O and O_2_. In this reaction, H_2_O_2_ acts as a substrate and hydrogen donor.

2H_2_O_2_→2H_2_O + O_2_ (catalytic reaction)

At low H_2_O_2_ concentration and with a suitable hydrogen donor, CAT acts as a peroxidant. In this reaction, the hydrogen donor is RH_2_ and could be alcohols, phenols, formaldehyde, or formic acid.

RH_2_ + H_2_O_2_→R + 2H_2_O (peroxidation reaction)

CAT is abundant in all aerobic cells and is mainly found in peroxisomes, the endoplasmic reticulum, cytoplasm, and mitochondria [140]. As far as the eye is concerned, CAT can be found in the cornea, AH, iris, radial body, lens epithelium, lens and retina [141].

##### GPx

The generic term GPx describes a family of isoenzymes containing selenium in their active region, such as the amino acid selenocysteine [142]. Four selenium-dependent GPx isoforms have been identified until now: (1) cellular or cytosolic or classical GPx (GPx-1), (2) gastrointestinal GPx (GPx-2), (3) extracellular or plasma GPx (GPx-3), (4) phospholipid hydroperoxide GPx (GPx-4). Selenium-containing GPx catalyzes alkyl hydroperoxide (ROOH) reduction in animal cells by consuming GSH, representing an oxidative damage inhibition mechanism. GPx-1 reduces H_2_O_2_ and various ROOH to H2O and alcohols using GSH as the electron donor. As a result of these reactions, two GSH molecules are oxidized to form GSSG. GPx-1 competes with CAT for H_2_O_2_ as a substrate and is a significant source of protection against low levels of OS [143]. Similarly, GPx-2 rapidly reduces H_2_O_2_ or fatty acid hydroperoxides. GPx-3 has a weak reducing effect on the cholesterol hydroperoxides, and GPx-4 effectively reduces the phospholipid hydroperoxides but weakly the H_2_O_2_ [142]. In the eye, GPx is found in the cornea, AH, iris, radial body, lens epithelium, lens and retina [144,145].

#### 3.3.2. Non-Enzymatic Eye Antioxidants

Non-enzymatic antioxidants can be hydrophobic molecules found in lipoproteins and membranes such as vitamin A, carotenoids and vitamin E, and hydrophilic molecules located in the cytoplasm, and mitochondria and nucleus, such as vitamin C and GSH [146]. Several studies have linked PEXS pathophysiology and progression to non-enzymatic antioxidants [33,38,42,63,74,83,95]. For instance, impaired retinoid metabolism is involved in the pathophysiology of PEX syndrome [63].

##### Vitamin A

Vitamin A belongs to a group of molecules having all-trans-retinol biological activity. Specifically, vitamin A contains retinal, retinol, and its esters, while provitamin A contains α- and β-carotene and β-cryptoxanthin, as these are the main nutritional precursors of vitamin A which are converted endogenously by enzymes to retinol. The preformed vitamin A is obtained exclusively from animal sources, while carotenoids are obtained from plant sources [147]. A relatively small number of carotenoids are found in humans, including cyclic ones, such as α- and β-carotene and acyclic carotenes, phytoene and lycopene, and several xanthophylls such as β-cryptoxanthin, zeaxanthin and lutein [148]. Other biologically important forms of vitamin A are *cis-* and *trans*-retinal and retinoic acid. Vitamin A and its analogs, such as retinal, retinol acetate, retinol palmitate and retinoic acid, act as antioxidants inhibiting lipid peroxidation. In addition to lipid peroxyl radical (LOO^•^), retinol can also clear the potentially harmful root of the GSH. In descending order, the antioxidant activity of retinoids can be described as follows: retinol ≥ retinal ≫ retinyl palmitate > retinoic acid [149].

Vitamin A plays a vital role in the antioxidant protection of low-density lipoprotein (LDL) against its copper-induced oxidation [150]. It has also been shown to regulate the daily circadian expression and the activity of many antioxidant enzymes such as CAT, GPx and glutathione reductase (GR) [151]. Carotenoids are associated with free-radical clearance mechanisms and are excellent quenchers of ^1^O_2_. Their antioxidant activity occurs in low partial pressure O_2_ (PO_2_), while in higher pressures, they can have pro-oxidative activity [152]. Carotenoids react with free radicals through different mechanisms, which depend mainly on their chemical structure, the medium’s polarity and the radicals’ activity. The three main reaction mechanisms are electron transfer, dehydrogenation and radical addition [153]. The concentration of carotenoids varies in the ocular tissues. Some are detected in traces, while others, e.g., lutein and zeaxanthin, are found in high concentrations in tissues, such as iris, radial body, lens and retina [154].

##### Vitamin E

Natural vitamin E is a mixture of eight fat-soluble components: four tocopherols and four tocotrienols (α-, β-, γ- and δ-), respectively. It is an essential element of the lipid bilayer of biological membranes with antioxidant and non-antioxidant functions. Vitamin E isomers are capable of delivering their phenolic ring hydrogens to free radicals, thereby exhibiting antioxidant properties. Based on the chemical construction of both tocopherols and tocotrienols, the hydrogen supply availability can be described in descending order: α > β > γ > δ. Thus, α-tocopherol (TOH), having three methyl groups, shows the highest antioxidant activity among tocopherols. Tocopherols protect cell membranes from photo-induced oxidative damage by removing ^1^O_2_ and other active primers, mainly by two mechanisms. Firstly, they act as “sacrificial chemical scavengers”, removing ^1^O_2_ in vivo, and secondly, they can act as natural deactivators or quenchers of ^1^O_2_ through a charge transfer mechanism [32,155,156].

Vitamin E is a significant fat-soluble antioxidant in cell membranes inhibiting lipid peroxidation. The process can be accomplished in two ways: either via clearing active forms (^1^O_2_, O_2_^•−^, OH^•^ and hydroperoxyl radical (HO_2_^•^)) that may initiate lipid peroxidation or stop lipid peroxidation if it is ongoing. The primary antioxidant role of vitamin E is to inhibit the spread of lipid peroxidation [88]. Vitamin E is mainly synthesized in plants and is an essential nutrient for animals and humans. In the eye, vitamin E is present in the AH, lens and retina [156,157,158].

##### Vitamin C

Vitamin C (L-ascorbic acid, AscH_2_, AA) exists in various forms depending on its oxidation state and the pH of the medium. Typically, most tissues dissolve 99.95% of vitamin C as ascorbate monoanion (AscH^−^). AscH^−^ at typical concentrations acts as a potent, water-soluble antioxidant that neutralizes a large amount of ROS and regenerates other smaller antioxidant molecules such as vitamin E. AscH^−^ is a very effective reducing agent and acts as a free-radical scavenger, efficiently providing an electron to various radicals, such as OH^•^, alkoxyl radical (RO^•^), LOO^•^, glutathionyl/glutathiyl radical (GS^•^) or TO^•^ [159]. An essential function of AscH^−^ is inhibiting lipid peroxidation and preserving vitamin E. AscH acts as a co-antioxidant with vitamin E, helping protect membranes from oxidative damage caused by LOO^•^. Moreover, it works synergistically with vitamin E, participating in the regeneration of the radical of vitamin E [160]. Humans, other primates, guinea pigs and a few species of fruit-eating bats ingest vitamin C mainly from plant-origin foods. Ascorbic acid in the eye can be detected in the cornea, AH, lens, vitreous humor and retina [161,162].

##### GSH

**Figure 5:**
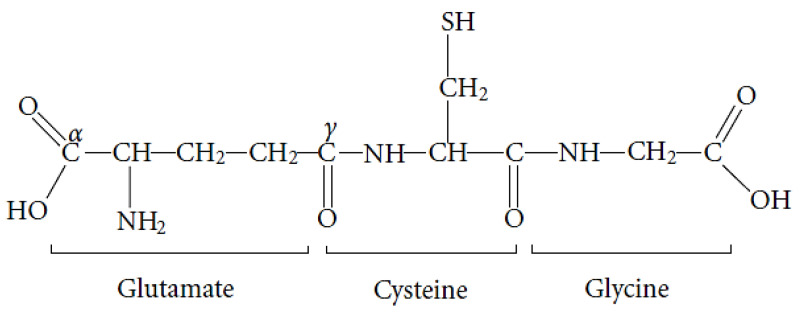
The reduced form of glutathione (GSH).

GSH is the major non-enzymatic endogenous antioxidant of the human organism involved in the cellular portion of the antioxidant protection system and represents the first defense mechanism against OS. GSH is the reduced glutathione, the enzymatically formed tripeptide L-γ-glutamyl-L-cysteinyl-glycine (L-γ-Glu-Cys-Gly) composed of the amino acids L-glutamic acid, L-cysteine and glycine (Figure 5). GSH is the major non-protein thiol in mammalian cells and is found intracellularly in millimolar concentrations (0.5–10 mM) while in blood plasma in micromolar concentrations [163]. It is synthesized exclusively in the cytoplasm, where it is mainly located (85–90%), but 10–15% of it is distributed in the extracellular space and different intracellular organs, such as the nucleus, mitochondria, endoplasmic reticulum and peroxisomes [164,165].

**Figure 6:**
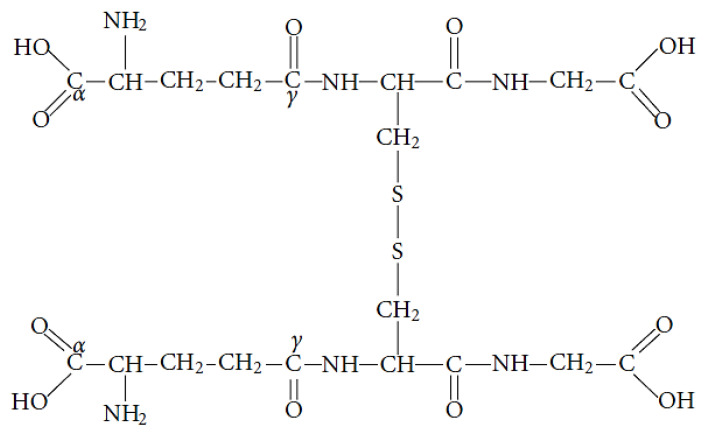
The oxidized form of glutathione (GSSG).

Cellular total glutathione may be free or bound to proteins. Free glutathione is mainly found in reduced form (GSH), which under OS, can be converted to an oxidized form, called glutathione disulfide (GSSG) (Figure 6).

GSSG returns to its reduced form through a reaction catalyzed by the enzyme glutathione reductase (GR), as follows:

GSSG + NADPH + H^+^→2GSH + NADP^+^

Of all redox couples that help maintain the redox environment of the cell, the GSSG/2GSH pair is the most abundant. Thus, the redox state of GSSG/2GSH is considered an indicator of the redox environment of the cell. Typically, in cells, the ratio [GSH]/[GSSG] exceeds 100, while under OS, the ratio decreases between 10 and 1 [165].

GSH plays a crucial role in the antioxidant defense of the cell. Its antioxidant properties are due to the reversible oxidation of the sulfhydryl group (−SH), which is in its active region. GSH is involved in both the direct (non-enzymatic) and indirect (GSH-associated enzymes) deactivation of active species [166].

GSH is involved in detoxifying end products of lipid peroxidation, e.g., MDA and 4-hydroxynonenal (HΝΕ), and many other products from the interaction of ROS with cellular components. GSH can also be covalently linked to proteins through S-glutathionylation. In addition, the antioxidant activity of GSH is exerted through its participation in the basic antioxidant systems of the cell. GSH helps maintain vitamins C and E in a reduced/active form through GSH-dependent enzymes that use it as an electron donor. Moreover, GSH detoxifies cells from peroxides through the action of GPx [167].

GSH is found in many foods but is synthesized endogenously by many cell types. Therefore, GSH is not a vitamin and is not an essential nutrient. The amino acids glycine, cysteine and glutamic acid are formed as products of cellular metabolism and are also absorbed through food [95]. GSH is detected in the cornea, AH, lens epithelium, lens and retina. GSH is essential for keeping lens proteins in a reduced state and, along with ascorbic acid, comprises the primary defense mechanisms against photooxidation [168].

## 4. Conclusions

An imbalance between antioxidants and oxidants plays a vital role in the onset of PEXS and its progression in humans. Recent genetic studies have identified several genes that contribute to PEXS/PEXG. Nevertheless, further research is required to provide additional insight into the molecular pathogenesis of the disease. The OS controls the remodeling of the ECM and modifies the antioxidant system so as to favor PEXS and its evolution into PEXG. To control and treat PEXS, it is essential to understand the pathogenetic mechanisms and elucidate the OS-mediated triggers. By understanding the contribution of antioxidants to PEXS, we will be able to develop novel therapeutic approaches for treating the disease in its early stages. The current management recommendations for PEXS involve increasing antioxidant intake. Specific dietary antioxidant supplements are used in patients with PEXS, albeit their effectiveness is limited. Therefore, there is a need for novel methods to be developed to reduce OS in PEXS patients.

## Figures and Tables

**Table 1:** Summary of eye antioxidants.

Eye Antioxidants	
Enzymatic	Non-Enzymatic
SOD	Vitamin A Vitamin E
CAT	Vitamin C GSH Cysteine
GPx	Carotenoids

SOD: superoxide dismutase; CAT: catalases; GPx: glutathione peroxidase; GSH: glutathione.

## Data Availability

Not applicable.
